# Gynecomastia in a Boy with Chronic Myeloid Leukemia during Imatinib Therapy

**DOI:** 10.4274/TJH-2013-0109

**Published:** 2013-09-05

**Authors:** Betül Tavil, Sibel Kınık, Ahmet Gözen, Lale Olcay

**Affiliations:** 1 Ankara Oncology Research and Training Hospital, Unit of Pediatric Hematology, Turkey; 2 Başkent University School of Medicine, Department of Pediatrics, Unit of Pediatric Endocrinology, Turkey; 3 Ankara Oncology Research and Training Hospital, Unit of Radiology, Turkey; 4 Ankara Oncology Research and Training Hospital, Unit of Pediatric Hematology, Turkey

## TO THE EDITOR

Chronic myeloid leukemia (CML) is a rare disease in children, accounting for 2%-3% of leukemias in this age group. Current treatment options of CML include tyrosine kinase inhibitors (TKIs) and allogeneic hematopoietic stem cell transplantation (HSCT) for children. Allogeneic HSCT has been recommended following attainment of remission with TKIs. Since long-term outcome and adverse effects of TKIs are uncertain in children, some authors have recommended HSCT within 2 years of treatment with TKIs [1,2,3]. Imatinib mesylate is one of the TKIs; it inhibits all ABL tyrosine kinases and selectively suppresses the ATP-binding site of platelet-derived growth factor receptor (PDGF-R) and c-KIT, which are expressed in various types of neoplasms and also in normal cells. It is well tolerated with mild adverse effects. Common and usually mild side effects of imatinib mesylate include edema, muscle cramps, skin rash, and conjunctival inflammation. An altered bone and mineral metabolism, a reduction of testosterone, and gynecomastia are the other defined side effects of imatinib mesylate, which were attributed to the PDGF-R and c-KIT inhibition in normal cells by imatinib mesylate. Gynecomastia development during the course of imatinib mesylate treatment has rarely been reported in the English literature [4,5,6,7]. Herein, we have described a 14-year-old boy with CML who developed gynecomastia while receiving imatinib mesylate for nearly 1.5 years. 

**Figure 1:**
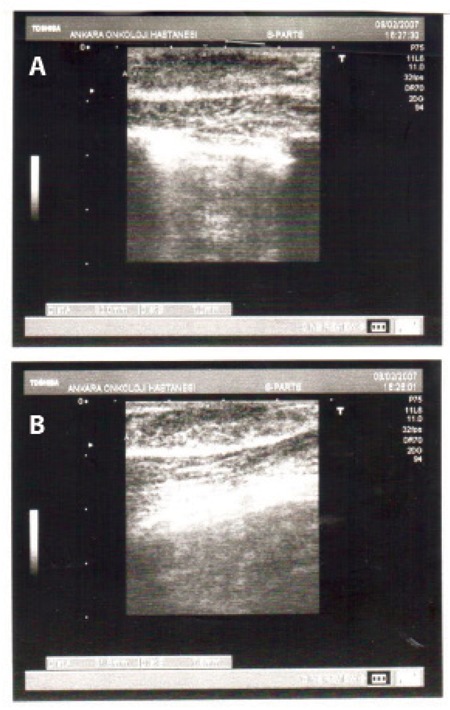
a) Breast tissue of 32x7.5 mm on the right side byultrasonography. b) Breast tissue of 30x7 mm on the left side

A 14-year-old boy was admitted to hospital with complaints of malaise, cough, and pain in the lower extremities. His previous medical history was unremarkable. There was no consanguinity between parents and he had 2 healthy siblings. On his physical examination, he had mild hepatomegaly (2 cm below the costal margin) and massive splenomegaly (18 cm below the costal margin). His laboratory studies revealed that his hemoglobin was 100 g/L; hematocrit, 23%; white blood cell count, 365x10^9^/L with 62% neutrophils, 14% stab cells, 6% metamyelocytes, 4% myelocytes, and 14% lymphocytes; mean corpuscular volume, 87 fL; and platelet count, 415x10^9^/L. Biochemical parameters were all normal. Studies for Epstein–Barr virus, cytomegalovirus, herpes viruses, toxoplasmosis, measles, mumps, rubella, parvovirus, Salmonella, and Brucella did not show any ongoing infection with these agents. His abdominal ultrasonography revealed hepatosplenomegaly. His echocardiography was normal. Bone marrow aspiration smears revealed hypercellularity and hyperactivity in the myeloid lineage with less than 5% blasts. Cytogenetic study of his bone marrow showed clonal positivity for t(9;22). Thus, the patient was diagnosed with CML in the accelerated phase. After the diagnosis of CML, he received cytosine arabinoside (150 mg/m^2^/day) and mitoxantrone (10 mg/m^2^/day) 2 times at a 1-week interval. Hydration (3000 mL/m^2^/day and 20 mEq/L, NaHCO3) and allopurinol treatment were continued until his leukocyte count decreased to normal levels. After learning that t(9;22) was positive, imatinib mesylate treatment (260 mg/m^2^) was started. Complete hematological response was achieved after 1 month of therapy. His t(9;22) was still positive after 3 months of imatinib mesylate. Complete molecular response was achieved 9 months after the diagnosis. While he was receiving imatinib mesylate treatment, a human leukocyte antigen-identical sibling donor was found for HSCT. However, his father refused the HSCT and the patient and his father preferred continuation of drug therapy (imatinib mesylate). At month 17 of imatinib mesylate therapy, he was found to have developed bilateral gynecomastia. His pubertal development was not completed at diagnosis (Tanner grade 1), whereas he was in puberty when gynecomastia was diagnosed. His β-human chorionic gonadotropin and α-fetoprotein levels were normal. His free testosterone level was decreased (3.48 pg/mL, normal range: 12-30) and his estradiol level was increased (25.69 pg/mL, normal range: 2-18). Bilateral breast ultrasonography also showed gynecomastia (Figures 1a and 1b). After he developed gynecomastia, he was irregularly admitted to the hospital for follow-up visits. When he presented to the hospital due to an accelerated phase of CML, it was learned that he had discontinued imatinib therapy for the last 8 months. It was found that the gynecomastia had resolved. Imatinib mesylate treatment was started again and complete hematological and molecular responses were achieved. During the consecutive 1-year follow-up, gynecomastia did not recur. However, later it was learned that he irregularly took the imatinib mesylate, and he was then lost to follow-up. Informed consent was obtained.

Imatinib is a signal transduction inhibitor; it inhibits the oncogenic tyrosine kinase BCR-ABL, c-KIT, and PDGF-R. c-KIT and PDGF-R are receptors of tyrosine kinases expressed in the testis and they are involved in testosterone production. These receptors are inhibited by imatinib mesylate treatment. This inhibition may be responsible for the development of gynecomastia. Gambacorti-Passerini et al. [7] reported that 7 of 38 men receiving imatinib for CML developed gynecomastia. They also measured hormone concentrations in 38 men receiving imatinib for CML at baseline and during treatment. The mean follow-up duration was 23.6 months in that study. A comparison of hormone concentrations in 21 patients before and during treatment showed that patients who developed gynecomastia had a reduction in free testosterone concentrations [7]. Liu et al. [4] reported that among 57 male patients treated with imatinib mesylate due to advanced or recurrent gastrointestinal stromal tumor, 6 (10.5%) developed gynecomastia during the treatment. Their serum free testosterone levels were within the normal range, whereas 3 had increased serum estradiol level [4]. Thus, reduced free testosterone levels and increased serum estradiol levels may be seen in patients receiving imatinib mesylate. Imbalance of sex hormones may be the cause of gynecomastia during treatment with imatinib mesylate. Free testosterone level was decreased and serum estradiol level was increased in our patient. At diagnosis, his pubertal development was not completed, whereas his puberty was completed during treatment and gynecomastia developed after puberty. We think that the gynecomastia resolved since the patient discontinued therapy first and then did not take it regularly during the consecutive follow-up period. In conclusion, boys who receive imatinib mesylate treatment should be examined from this aspect during follow-up visits. It should be kept in mind that gynecomastia can develop in a boy with CML following long-term imatinib mesylate treatment.

## CONFLICT OF INTEREST STATEMENT

The authors declare that they have no conflicts of interest that could be perceived as having influenced the impartiality of the materials presented.

**Funding**

The present study received no grant from a funding agency in the public, commercial, or for-profit sectors.
